# What Shall the Surgeon Do with the Urethra and the Testes in Cases of Amputation of the Penis

**Published:** 1889-02

**Authors:** W. Locke Chew

**Affiliations:** Birmingham, Alabama


### WHAT SHALL THE SURGEON DO WITH THE URETHRA AND THE TESTES IN CASES OF AMPUTATION AND ABLATION OF THE PENIS.*

BY W. LOCKE CHEW, B. S. M. D., BIRMINGHAM, ALABAMA.

Mutilation in surgery is criminal. Removal of organs to insure physiological rest to another disease is just and right. The removal of organs to produce changes in nutrition in associate organs, where function is prevented or not desired, should be attentively considered. The removal of organs to produce mental quietude and diminish physiological desires, after the ablation of associate members, is not unworthy of careful thought.

Surgical errors are often committed, and surgical teaching

*Read before the Medical Association of the State of Alabama.

often warped from lasting truth by the clinicians having preconceived ideas on the subject, then constructing (may be unconsciously) his case-history to suit the idea; hence, in a given number of cases, the observation being wrong, the deduction is erroneous, and it takes years to correct these conclusions. In the preparation of this paper this has been carefully avoided, and no desire on any side has changed any fact from the clinical observation, but desiring to illustrate clearly certain points, material has been drawn from journalistic articles, and not wholly from my note-book, so that the cases given can be regarded as the true clinical record of various medical observers.

The subjects on which these cases bear, are best set forth in the following question: What shall the surgeon do with the urethra, and the testes, in case of amputation and the ablation of the penis? This question is one of moment to the surgeon, who assumes the responsibility of being the surgical adviser of a patient suffering from sarcomatous, or carcinomatous growths of the penis—diseases tending to early death. What should be done? Should an attempt be made to save the greater part of the organ, risking recurrence and jeopardizing life, or should the corpora cavernosa be removed in toto from their attachments, and the lymphatic glands be removed from the groin? Mr. Gould has shown the great liability to recurrence in the corpora cavernosa, and other surgeons the likelihood of the extension through the lymphatics and the blood currents.

The per centum of recurrences in the corpora cavernosa would appear to be sooner or later 97, and if accurately known it would most probably recur in each instance. Recurrences in the corpus spongiosum can not be had, but it would appear very low, if at all, until it recurred in the cavernosa. In carcinoma of the penis, here, as in other localities, it is found that the lymphatics are involved, no matter what the stage, in about 95 per cent. of all cases, and should be removed at earliest date. The per cent. of extension of sarcoma through the blood currents can not be stated, though it is here about as in other cases. The subject divides itself into two classes, according to the extent of invasion of the disease, for in each case does the operative procedure differ in regard to the urethra, and advice in regard to the testes.

First stage.—Where coition shall be possible after total removal of the diseased tissue of the corpora cavernosa.

Second stage.—Where coition shall be impossible after ablation of the organ.

First stage considered.—Where the disease exists, as the ulceration button confined to the prepuce, then it is judged wise to do a wide-reaching circumcision, removing every part of the foreskin and the inguinal glands on either side. The chances are that the disease may be limited, and the patient recover. Some few have been known in whom the disease never afterward recurred. This is too infrequent to rely upon. Again, if the disease is beginning to infiltrate the glans penis, or corpora cavernosa, what shall be done? If the patient is such an age that there is circumstance for the function of the organ, it is judged wise to do an amputation well away from the diseased tissue, yet saving all possible—enough for coition—and removing at the same time the inguinal glands thoroughly. The testes should be allowed to remain undisturbed. What shall be done with the urethra? How shall retraction and contraction be prevented? Before proceeding further, however, consider the various methods heretofore employed, with their relative merits and demerits, and the anatomical attachment of the urethra in the normal penis.

1. Ricord's old method.—That of slitting the urethra into three or four parts, and attaching these by means of interrupted *sutures* to the skin. To this method there are objections. (*a.*) Difficulty of proper union between the urethra and the skin. (*b.*) Does not permit the entire stump to be covered at every point, hence, suppurative, cicatricial inflammation. (*c.*) Does not prevent retraction. (*d.*) Does not prevent obstruction.

2. Watson's (Edinborough) method.—In this the urethra is brought out below through the integument, and nearer the scrotum, this is to prevent stenosis of the meatus by removing it from the cicatrizing process necessary for the healing of the stump. Objection (*a.*) Micturition unsatisfactory, meatus opening downward. (*b.*) In short stumps coition is unsatisfactory, and impregnation difficult, the semen being frequently lost. (*c.*)

Does not prevent retraction, which (*d.*) causing inversion of the integument produces meatal stenosis.

Humphries' and Hintons' modifications have no points of recommendation worthy of special note. Looking next at the urethra, in the normal penis and glans, how do we see retraction prevented ? The corpora spongiosum is broadened out into a wide, thick expansion, the glans, which is fitted over the ends of the corpora cavernosa. This prevention of retraction of the urethra is, as I consider it, one of the functions of the glans, and was first called to my attention by a case of gonorrhœal chordee, in which so great was the urethral inflammation and the traction on the attachment of the urethra, that it caused marked curvature of the corpora cavernosa. To learn from this observation, if any thing can be drawn from one notation, it would appear that the urethra should be attached to the corpus spongiosum, and the corpus spongiosum carried in a broad expanse over the amputated ends of the corpora cavernosa, which gives it a strong support. The vigorous attack made by the disease renders it necessary, too, to divide the corpora cavernosa an inch beyond the point of the apparent infiltration and the freedom from involvement of the corpus spongiosum, enables us to save a long lower flap which contains the urethra. The cuticle of this flap should have a wedge-shape piece next removed as follows: In the centre of the flap remove from the skin a wedge-shaped portion, the base of the wedge being directed toward the end of the flap, half-inch from it, the apex directed towards the scrotum, about half-inch from the base of the wedge. Through this wedge-shaped aperture should next be drawn the urethra, which will project some half-inch beyond the cuticle, next split the urethra along its under surface, from the cuticle to its cut extremity, and suture the cut end of the urethra to the base of the wedge-opening in the cuticle of the flap, and the slit margins of the urethra to the edges of the wedge-opening, so that accurate adaptation and approximation is effected. Next, the hemorrhage being entirely checked and the oozing stopped, suture with Kocher-gut the raw surface of the flap to the divided ends of the corpora cavernosa, and by interrupted sutures unite the edges of the flap to the cut edges of the cuticle of the stump. The cut ends of the corpora cavernosa act as a prop and splint to the corpus spongiosum as in the normal organ. The objection that confronts us in this method is a simple one, only that of securing ready union between the cut ends of the stump, and the raw surface of the flaps. This is difficult, because oozing is so liable, and it is difficult to apply a permanent sublimate dressing. I think it wise, however, to leave a catheter in, during the first few days after the operation, and let it pierce the dressing which should be kept aseptic for ten days by re-applying if necessary with great precaution. The patient thus operated on should not be pronounced relieved until after three years, for Gould has shown the great liability of recurrences in the corpora cavernosa and the danger from lymphatic absorption, and recurrence is already known. Therefore, it is advised that the patient be kept under the strictest course of observation, prepared at any time to undergo the more radical ablation of the corpora cavernosa.

*Second Stage.*—Such is the conclusiveness of surgeons, and such their power of actual demonstration of certain facts in regard to this stage of the disease, that the law is marked out, and the surgeons of to-day have but to act. Mr. A. Pearce Gould has shown that the recurrence is in nearly every case in the corpora cavernosa, hence they should be removed from their attachments. Prof. Humphries, of Cambridge, first suggested and practiced, and afterwards Thiersch demonstrated the great advantages to be derived from transplantation of the urethra to the perineum, advantages apparent to the patient and surgeon, excoriation from urine being prevented and danger from urethral retraction lessened.

Mr. Gould's modification also, that of splitting the scrotum and and the removal of the cavernous bodies from their attachments to the ischia and pubes is to be regarded as a great advance, and is founded on accurate, scientific and surgical principles.

(*a*) Disease is much less likely to recur.

(*b*) Gives ample room for removal of the cavernous bodies.

(*c*) Enables thorough drainage and prevents bagging of pus and serum.

(*d*) Removes the fear of contraction and retraction of the urethra.

(*e*) Does not increase danger from hemorrhage.

(*f*) Fatality increased but little.

The following case of Jassett's illustrates the Gould modification of the Humphrey–Thiersch operation; and while in the aged and infirm, where vitality was but slight, and with all the complications expected, yet the result is perfect.

EPITHELIOMA OF PENIS.

BY FRED B. JASSETT.

*London, England, Lancet, May 22d,* 1886.

*Case.*—J. W.; æt. 70. Admitted to Cancer Hospital December 5th, 1885. Compositor. Family history excellent, except father's mother died of cancer of stomach. No specific history. Liquor drinker. Seven months ago noticed an increasing, red, painful ulcer on lateral aspect of penis, midway the organ. Suffered greatly from chronic bronchitis. Large fungating and offensive growth on penis extending to root. Three days later, the patient being etherized and in the lithotomy position, with pubes shaven, a circular incision was made around the root of the organ, and another incision extended from the lower part of this along the line of the raphe of the scrotum, well into perineum, and the two halves of the scrotum separated quite down to the root of the penis." The corpus spongiosum was carefully dissected from the corpora cavernosa as far back as the triangular ligament.

The corpora cavernosa were next exposed as far back as the rami of the penis, when with a periosteal raipistory they were severed from their attachments. The urethra was split for half-inch, and stitched to the inferior angle of the wound. Dressing, iodoform, drainage tube, and continuous suture of horsehair. Slough about centre of wound on the 15th; scrotum ecchimosed and inflamed. On the 30th day, urinary abscess from infiltration of urine formed in the perineum. Incised, urine passing out. Urethra retracted, urine passing freely. Fistula closed soon. Restored to health completely.

Does removal of the penis cause melancholia, or associate mental and exaggerated sexual phenomena, and what would appear to be the best means of remedying them? In the study of the question, it does appear from the history of recorded cases, that total, or even partial removal of the penis, might cause marked mental and physiological discomfort. For the relief of the physiological expressions, when they occur painfully frequent from an erotic temperament associated with an ever hopeless inability for the sexual act, an early castration promises a speedy cure. In the study of the whole number of cases in regard to relief of the mental disturbance, I fail to find any record which without doubt bears on the question. The cases of Mr. Wheelhouse approach nearer than any other I have found, yet they do not show mental discomforts relieved by castration. They show only in case of castration, with ablation of the penis. These annoyances fail to appear. (This is the rule in most cases when the testes remain.) Another point worthy of note is, that there are no recorded cases (as far as I have found) of melancholia or other troublesome mental phenomena, sequelæ of castration or atrophy. The dangers of castration add so little gravity to the operation, that could it be shown that it was curative it would be advised that this additional danger be incurred. The following, bearing on this question, although not positive except concerning the physiological desires, yet are of sufficient interest to justify further record or study.

MR. WHEELHOUSE'S (LEEDS, ENGLAND) CASES.

These two cases were operated on at the same time, and occupied adjoining beds in the hospital. In one, the disease had invaded the testes, and they were removed along with the penis, the urethra being transferred to the perineum. A similar operation was done on the other, except that the testes not being involved, were undisturbed.

"The one in which the testes were removed, made an uninterrupted recovery, and expressed himself as feeling completely relieved in every way; the other, however, although he made a good recovery, was a martyr to the physiological action of his testes, which were a constant source of annoyance to him, and he still remains a prey to desires he can never gratify. I think every one will admit that the condition of the former patient was certainly more satisfactory and perfect than the latter. This, perhaps, is one drawback to the special form of operation now being discussed, as by the old method, so long as any portion of the penis remained, the question of removing the testes need not arise, On the other hand, however, I am strongly of opinion that the immunity from recurrence in the stump is of far more importance than this minor inconvenience to the patient. Therefore, in performing amputation of the penis in men under fifty years of age, this point should be fully laid before them, and as risk to life by loss of the penis is not materially increased, we should rather advise their removal." (Dr. Jassett, Lancet, 1886.)

CASE—AMPUTATION OF THE PENIS.

DAVID JAMISON, M. D.

*New Orleans Medical and Surgical Journal, 1886.*

A. T., æt. 36. Creole. A cooper, who never had a venereal disease, presented at the Surgery February 1st, 1886, with a sloughing glans penis, which had been often traumatised in his daily occupation. He "had a long prepuce which had ulcerated away." No hereditary tendencies. Inguinal glands not enlarged. "He begged that as much of the organ as possible be saved." Section showed epithelial cancer. Under chloroform, penis amputated about middle, and mucous membrane stitched to skin. (Ricord.) Wound united kindly before tenth day. Three months later returned with fungated growth extending to root of organ, inguinal glands on either side involved. Second operation under chloroform, corpus spongiosum divided three inches in front of bulb. "The urethra was dissected, from the body of the penis, as far back as possible. The body of the penis was now divided, and the urethra left projecting from the wound." Wound cured rapidly. "One point of interest in this case is the effect produced on the patient's mind by the removal of the penis.

After the second operation, when he found the organ entirely gone, he became morose and melancholy, and lost his appetite. He sat in one position, and took no interest in his surroundings."

Remarks.—This case shows how dangerous it is to leave the inguinal lymphatics, even though not involved, for in 117 cases of cancer the microscope showed enlargement of the glands in 115 (Keaster). The case also shows how great may be the mental disturbance in the warm-blooded and the young, after ablation of this organ. The mental phenomena only appeared after the total removal of the organ. Is it not justifiable to infer that the patient, aged 36 years, had not lost the sexual appetite, for he begged "that as much of the organ as possible be saved?" Is it not permissible, too, to infer that if this disease was removed, the melancholia would have disappeared? In other words, that the ungratified physiological expressions were the cause of the melancholia? Might not castration have been suggested with a hope here of relief? In the five cases which have come under my observation, in each has there been retraction of the urethra. Of these, four were by Ricord's and one by Watson's method. Stricture of the meatus occurred in four cases after the fifth month. One by Ricord's method suffered from a painful stump, as did the one by Watson's method. The pain of the latter seemed due to lateral expansion of the organ and stretching of cicatrix and not by elongation of the organ. In one case the patient urged that enough of the organ be saved for copulation, for he had been driven to seek the operation by fear of disloyalty on the wife's part. Fertilization is thought to have taken place within fifty days after the operation, with thorough adjustment of all differences. In England, the Gould modification is followed. Dr. Wyeth finds the original plan of Humphries satisfactory. All operators report the transplantation of the urethra to the perineum a success. In two instances in the above five cases, in which the disease is known to have recurred, recurrence was in the cavernosa first. The third case met with a sudden death. The fourth was lost sight of, and in the fifth the disease has not returned, seven months having elapsed. Dr. Boone recommends (Lancet, Oct. 1st, 1887), a modification of

Gould's method, by a division of the operation into two operations, forming a new urethra prior to the removal of the cavernosa. In the very feeble, this is a distinct gain, lessening the danger from the urethra at time of the principal operation. What then shall we say in conclusion of the whole matter?

1st. That were the desire to perpetuate life only, we should advise in each case of advanced sarcoma or carcinoma of the penis, immediate removal of the corpora cavernosa, down to the rami of the ischia (Gould), simultaneous castration being laid before the young, and enucleation of the inguinal and perineal glands in every case, together with the transferal of the urethra to the perineum. (Humphrey-Thiersch). But considering the fact that it is always desirable to save the penis and testes, it is advised,

2d. That as long as such a portion of the organ may be saved that sexual congress can be effected, procreation possible, that the testes should be undisturbed, that the urethra should be brought out of the end of the penis, slitting the urethra and stitching it to the perforated slit in the center of the lower flap, allowing the newly formed meatus to be involved as little as possible in the contracting inflammation.

3d. That such are the mental phenomena liable to follow total ablation of the penis, in those of strongly marked sexual affinity, that it should not be ignored, but that this fear on part of the surgeon should never make him advise simultaneous castration, for these phenomena are rare, and castration can be readily resorted to at any time, but as a means of relief in these cases is worthy of trial.

4th. That in malignant diseases of the penis, the statistics of recurrence in the testes does not warrant castration or removal of the scrotum, for this alone, but invasion of either having begun, the total removal of both should be advised.

5th. That where total ablation is called for, that the urethra should be transferred to the perineum (Humphrey and Thiersch), with the testes, and scrotum preserved for future operation, should symptoms or extension of the disease afterwards call for it.

